# Evaluation of the collaborative integrated surveillance system (ViCo) in Guatemala: a qualitative study on lessons learned and future perspectives

**DOI:** 10.1186/s12889-022-12719-7

**Published:** 2022-02-18

**Authors:** Jahn Jaramillo, Mariangeli Freitas Ning, Loren Cadena, Michael Park, Terrence Lo, Emily Zielinski-Gutierrez, Andres Espinosa-Bode, Marines Reyes, Maria Del Rosario Polo, Olga Henao

**Affiliations:** 1 Public Health Institute/U.S. Centers for Disease Control and Prevention Global Health Fellowship Program, Oakland, CA USA; 2 Division of Global Health Protection, Central America Regional Office, U.S. Centers for Disease Control and Prevention, Guatemala City, Guatemala; 3 Training Programs in Epidemiology and Public Health Interventions Network, Decatur, GA USA; 4 Division of Global Health Protection, U.S. Centers for Disease Control and Prevention, Atlanta, GA USA

**Keywords:** Evaluation, Surveillance system, Emerging infectious diseases, Implementation, Qualitative, Interviews, Guatemala

## Abstract

**Background:**

The collaborative integrated surveillance system known as *Vigilancia Integrada Comunitaria* (ViCo) was implemented in 2007 to better understand and characterize the burden of diarrheal, respiratory and febrile illnesses in Guatemala.

**Methods:**

To evaluate the usefulness of ViCo and inform a redesign of the system and new surveillance activities in the Central American region, personnel from the United States Centers for Disease Control and Prevention (CDC) conducted thirty-nine in-depth interviews from June—December 2018 with key stakeholders responsible for the design and implementation of ViCo in Guatemala. A semi-structured questionnaire adapted from the Updated CDC Guidelines for Evaluating Public Health Surveillance Systems was used for data collection. We used a grounded theory approach to explore stakeholder perceptions of ViCo and generate recommendations for improvement. Primary qualitative findings were organized based on thematic areas using ATLAS.ti version 8 software.

**Results:**

Emergent themes relevant to the usefulness of ViCo were organized across strengths, weaknesses, and recommendations pertaining to the: (1) Size and Complexity of ViCo, (2) Stakeholder Expectations About the Objectives of ViCo, (3) Data Management and Structure of the Information System, (4) Local Control of Data, (5) Integration of ViCo within the Ministry of Health, and, (6) Improvement of the Operational and Design Aspects of ViCo across System, Process, and Output levels.

**Conclusions:**

Stakeholders perceived ViCo to be useful. They recommended measures to improve system performance and quality, including simplifying the surveillance system, routine data analysis and feedback, and channeling efforts towards integrating surveillance data into the national health information system. To create a well-performing surveillance system and achieve the intended objective of surveillance for public health action, ongoing evaluation and assessment of surveillance activities are necessary.

**Supplementary Information:**

The online version contains supplementary material available at 10.1186/s12889-022-12719-7.

## Introduction

Public health surveillance is the continuous and systematic collection, analysis, interpretation, and dissemination of health-related data for public health action to improve health [1]. An effective surveillance system can provide early warning for public health emergencies and help guide the planning, implementation, and evaluation of public health activities and policies [1]. Periodic evaluations of surveillance systems are therefore essential for enhancing the quality, efficiency, and usefulness of information that drives informed decision-making in rapidly changing environments [1–3].

There is a need for evaluation data to guide the development of robust and useful disease surveillance systems that are also sustainable [4]. Understanding the extent to which surveillance systems are able to successfully collect and analyze data to estimate the size of a health problem in a population and document the distribution and spread of disease is critical to improving their effectiveness and their relevance [5]. Significant challenges in evaluating surveillance systems include identifying the benefits of surveillance and response systems and the costs involved with conducting systematic evaluations [1, 6]. To address these challenges, assessing the non-monetary costs and benefits of surveillance holds promise for generating valuable insights for decision-makers [7]. Despite their impact on system performance, non-monetary costs are rarely considered in evaluations, revealing a fundamental gap in the literature that requires attention to move the assessment of surveillance systems forward [6].

Additional research is necessary to understand the views of stakeholders responsible for the design and implementation of disease surveillance systems, including the strategies, strengths, weaknesses, and recommendations for improving surveillance efforts [7, 8]. Stakeholders play an active role in the definition and analysis of problems encountered during the implementation of surveillance activities. This expertise can be leveraged for finding solutions to these problems [8]. This evaluation assessed the Integrated Community Surveillance (ViCo) system in Guatemala, where emerging infectious diseases continue to be a significant burden [9], from the perspectives of stakeholders. We explored the multi-level factors and processes that shaped the implementation of the system. To evaluate the non-monetary benefits of the surveillance system, realistic and context-adapted recommendations were obtained from stakeholders to enhance the system's acceptability and guide future iterations of ViCo [5, 10]. We expect that results will improve future surveillance in Guatemala while also adding to the larger global discussion on how to best harness the benefits of surveillance activities.

## Background

To better understand and characterize the burden of diarrheal, respiratory and febrile illnesses in Guatemala, a sentinel surveillance system known as *Vigilancia Integrada Comunitaria* (ViCo), or the integrated community surveillance, was established in 2007. The Universidad del Valle de Guatemala (UVG), in collaboration with the Guatemala Ministry of Public Health and Social Assistance (MSPAS) and the U.S. Centers for Disease Control and Prevention (CDC), designed and implemented the facility-based syndromic surveillance system to generate high-quality data among a network of sentinel reporting sites, document the distribution and spread of disease, evaluate control strategies and interventions, identify research needs, facilitate national planning for surveillance activities, and lay the foundations for early warning of outbreaks and emerging infections.

Surveillance activities were carried out in the departments of Santa Rosa (population 320,000), located 50 km southeast of Guatemala City, and Quetzaltenango (population 705,000), located 120 km northwest of the capital, across three levels of health services (i.e., hospital, health center, and health post) from 2007–2018 [9, 11, 12]. Briefly, project-hired surveillance nurses reviewed registers and emergency department logs to identify and admit patients with acute infectious disease symptoms (e.g., diarrheal, respiratory, febrile). Clinical, demographic, and epidemiological data were obtained from face-to-face interviews with patients who met the case definition for the illnesses under surveillance and from chart reviews.

Depending on the syndrome, biological specimens were collected and tested for a variety of emerging pathogens of interest including but not limited to dengue, norovirus, rotavirus*, Campylobacter, Salmonella, Cyclospora,* influenza, respiratory syncytial virus*, Streptococcus pneumoniae* [11, 13–18]. Linked epidemiologic and laboratory data were shared with MSPAS and CDC on a weekly basis. The data collected were used to better understand the distribution and trends of the different syndromes under surveillance and reviewed to ascertain potential changes in occurrence or in populations being affected. Reports were generated and periodically shared among stakeholders, including the health facility and hospital epidemiology staff, and MSPAS officials. In addition to sentinel surveillance, ViCo was also used as a foundation for smaller-scale research studies which tapped into existing surveillance activities to evaluate interventions and diagnostics [19–21].

## Methods

### Conceptual framework

A logic model for ViCo (Additional file 1) was developed during the formative stages of ViCo to highlight the objectives and outcomes of the surveillance system inputs and activities. To assess the applicability of the logic model to the specific research objectives of ViCo, CDC’s Updated Guidelines for Evaluating Public Health Surveillance Systems were used as a framework to guide the development of the interview topic guide, as well as analysis of the data collected [1]. The updated guidelines included topic areas related to the integration of surveillance and health information systems, the establishment of data standards, the exchange of health data, and changes in public health surveillance objectives to facilitate the response to emerging health threats.

### Setting and participants

Stakeholders from CDC, UVG, and MSPAS who had worked in Guatemala and the United States during ViCo were selected purposively to reflect different organizational structures and levels, scientific agendas, degrees of research activity, and historical engagement across ViCo’s 10-year operation. Our target sample goal was 40 participants, which is consistent with qualitative research with heterogeneous samples to achieve data saturation [22]. A comprehensive list was compiled by CDC staff with the following inclusion criteria: current and past CDC, UVG, or MPSAS staff; considered a key informant with first-hand knowledge about ViCo and the community; direct involvement with and influence in the development of ViCo (e.g., protocol development, management/supervision, implementation); and worked directly with ViCo at any given point in time from 2007–2018. Those without current contact information were excluded from the list. Our sample consisted of the ViCo system users from across the program’s implementation period and included central, district and health-facility level MSPAS staff, university and field staff from UVG, and CDC staff from headquarters in Atlanta and in the Guatemala country office location. Stakeholders were instrumental in the design and management of the surveillance system at varying time periods and could offer a comprehensive view of the successes and challenges of implementing ViCo.

### Data collection

A semi-structured interview guide was developed based on prior research on the barriers and facilitators to implementing community-based surveillance systems and the CDC Updated Guidelines for Evaluating Public Health Surveillance Systems [1]. The interview guide was designed with an open framework to guide the interview, while allowing for a conversational format (Additional file 2). Interviews explored stakeholder perspectives of the surveillance system's objectives, the evolution of ViCo, the usefulness of ViCo, quality of data outputs, the impact of ViCo, and recommendations for improving ViCo. In-person or phone interviews were conducted with Spanish- and English-speaking stakeholders involved in the design and operationalization of ViCo; all were carried out by the second author (MFN), who was not involved in the design or the implementation of ViCo. To communicate their knowledge of ViCo and its major components, key informants were asked to visually represent their understanding of the ViCo surveillance system, including data pathways, from initial data collection to data dissemination. Notes were taken, and conversations recorded, with permission, using a hand-held digital recorder. Each recording and associated materials (i.e., participant drawings and interview notes) were assigned a study identification number. Written informed consent was received from each stakeholder interviewed. This activity was reviewed by CDC and was conducted consistent with applicable federal law and CDC policy.^1^ This evaluation was nested as an essential activity within the ViCo surveillance system study protocol which received IRB approval from CDC (#5150), UVG (#005–04-2008), and MSAPS (#18–2014).

### Data analysis

Notes and recordings from in-depth interviews were transcribed verbatim and translated from Spanish to English by the fifth and sixth authors (MR and MDRP). Interview transcripts were reviewed and anonymized, and any names or other individually identifying references were removed. Data were analyzed using framework analysis to ensure the validity, reliability, and replicability of qualitative research [23, 24]. The first author (JJ) and co-investigators (MP and TL) developed the initial analytical framework containing broad preliminary codes to define concepts (e.g., trends, evaluation, dissemination), which was supplemented by an inductive approach to identify themes emerging from the data, based on a Grounded Theory approach [23, 25]. For each construct that emerged from the data, the construct’s strength and consistency with the conceptual framework were explored by documenting the frequency, importance, and representativeness. The framework was subsequently refined through a reflexive and iterative process. The first and second authors (JJ and MFN) independently coded two transcripts, after which divergent coding was discussed, codes revised, and new codes added to the codebook. A subset of 10 interviews (with equal representation from CDC, UVG, and MSPAS stakeholder groups) were double coded by JJ and MFN, and inconsistencies were discussed among the coders to ensure coding reliability and consistency and add rigor to the coding process [26, 27]. JJ independently completed the final coding of all transcripts. ATLAS.ti qualitative software version 8 was used to manage and organize the interview transcripts [24]. Quotes from respondents are included in the text to highlight findings.

## Results

### Study population

From a total of forty-one interview invitations sent to stakeholders, thirty-nine (95%) in-depth interviews with key informants were conducted across three different stakeholder groups and at different levels (e.g., central, district, health facility) [UVG (*n* = 17), MSPAS (*n* = 12), and CDC (*n* = 10)] before it was assessed that saturation was reached (Table 1). Each interview lasted from 30 min to two hours. Participants represented epidemiologists (*n* = 14), directors (*n* = 11), laboratorians (*n* = 7), project nurses (*n* = 5), and informatics (*n* = 2) who were involved in the design and implementation of ViCo. Most participants were female (*n* = 24, 62%) and from Guatemala City (*n* = 24, 59%). Two stakeholders could not be reached via email or phone for an interview request.

**Table 1:** Demographic characteristics of stakeholders interviewed by gender, organization, position, and location

Study Participants Interviewed (*N* = 39)			
		**N**	**%**
**Gender**			
Female	**24**		**62**
Male	**15**		**38**
**Organization**			
UVG	**17**		**41**
University	9		**-**
Field	8		**-**
MSPAS	**12**		**33**
Central	7		**-**
District	3		**-**
Health Facility	2		**-**
CDC	**10**		**23**
Headquarters (HQ)	7		**-**
Country Office (CO)	3		**-**
**Position/Role**			
Epidemiologist (Epi)	**14**		**36**
Director	**11**		**28**
Laboratory	**7**		**18**
Project Nurse	**5**		**13**
Informatics (IT)	**2**		**5**
**Location**			
Guatemala City, Guatemala	**18**		**46**
Santa Rosa, Guatemala	**7**		**18**
Quetzaltenango, Guatemala	**7**		**18**
Atlanta, USA	**7**		**18**

### Emergent Themes

**Fig. 1:**
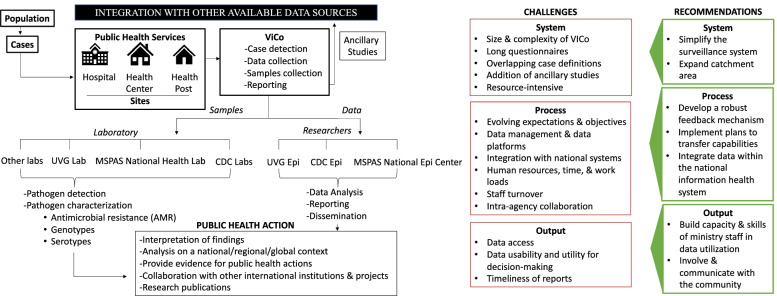
Perceived challenges and recommendations to improve ViCo across system, process, and output levels

Across interviews, key themes were organized to demonstrate the: (1) Size and Complexity of ViCo, (2) Stakeholder Expectations About the Objectives of ViCo, (3) Data Management and Structure of the Information System, (4) Local Control of Data, (5) Integration of ViCo within MSPAS, and (6) Improvement of the Operational and Design Aspects of ViCo across System, Process, and Output levels. (Fig. 1).

#### Size and complexity of ViCo

**Fig. 2:**
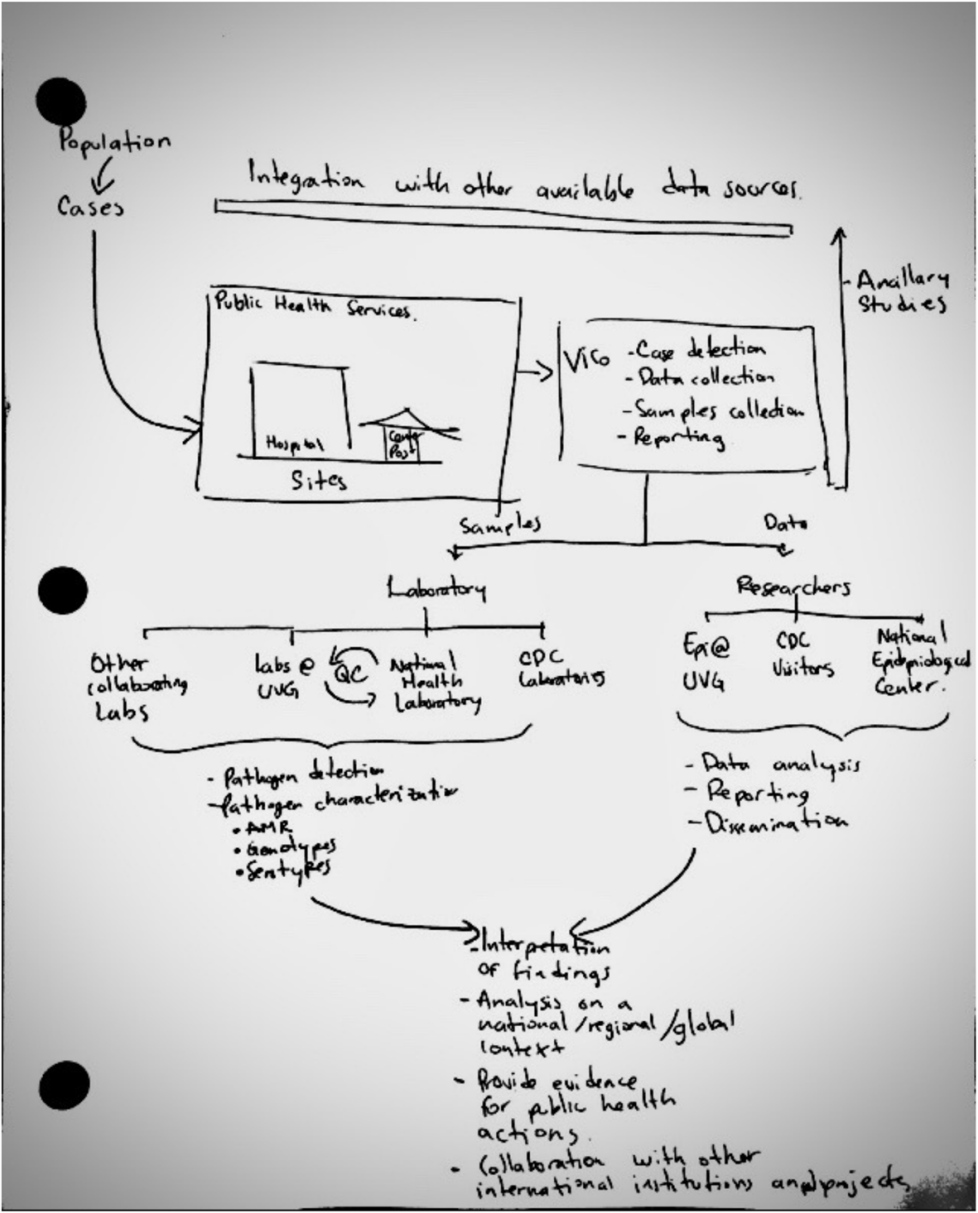
Selected UVG stakeholder hand-drawn diagram of the ViCo surveillance system illustrated during interview

When asked to draw a flowchart of ViCo processes, all stakeholders expressed a basic knowledge of the purpose and objectives of the surveillance system. Stakeholders from UVG gave the most “systems-focused” description, focusing on the organization and functionality, its major components (e.g., population, time period, data sources, what/how data were collected, policies and procedures regarding data management and privacy) and strengths including data management, dissemination, and analysis (Fig. 2). Stakeholders explained that weaknesses in the design and structure of ViCo were related to its complexity, often referencing the large amount of data elements collected for the priority diseases. The amount of epidemiologic, clinical and laboratory data collected under ViCo contributed to its size, the personnel needed, and the funding required to sustain it.

*VICO could have been much simpler. In the end we created a rather complex system that worked but did need sophisticated personnel or methods to be able to work, then replicating VICO or inheriting VICO by the Ministry of Health is quite a task ... It is quite expensive because it would mean transferring a lot of technology and knowledge and the Ministry believes that it could not sustain it, so another lesson... a system [that is] simpler.* **Participant (P)4, UVG Informatics, Guatemala**

Respondents in all stakeholder groups felt that the design of the patient interview questionnaire could be simplified as it included many questions that, in their perspective, were never completely used.

*The questionnaire was so long [and] it was of little use to the country. So much information that was collected... We never saw, at least I, the use of everything that was socioeconomic.* **P39, MSPAS, District Epidemiology, Guatemala**

*There were some questions that [patients] couldn't answer… the type of bathroom in their house because they don't like people [to] ask them those things.* **P33, Project Nurse, Guatemala**

#### Stakeholder expectations about the objectives of ViCo

According to participants from UVG, a key advantage that emerged from ViCo was the generation of a robust database that helped generate research publications and reports on both a national and international scale.

*[ViCo] databases have been used to generate publications. And… the data themselves, for the use of other people in public health.* **P27, UVG Director**, **Guatemala**

However, as the surveillance system evolved over the years, stakeholders alluded to changing expectations and objectives of ViCo. Respondents from MSPAS expressed their thoughts and concerns about whether the data ViCo was generating were used for research and surveillance purposes or to diagnose and treat patients.

*One thing that [we were] always criticized [for]…was that if we had the capacity for [ViCo], why didn't we use it for diagnosis… Primarily, the project was one of surveillance. But, independently, we sometimes use[d] it... as a diagnosis and to define therapies... more than anything in children.* **P34, MSPAS District Director, Guatemala**

CDC stakeholders observed that the addition of ancillary studies contributed to a sense that ViCo’s original objectives had changed and that the working parts of the system were becoming increasingly difficult to manage. While respondents perceived the integration of short-term research studies to generate useful data, it was unclear whether it was necessary to continue collecting this data as part of the core surveillance activities, even after smaller scale studies concluded.

*This is a platform… [where] you can set up short term studies … I think whereas ViCo started as a surveillance platform… it evolved more over the years… it moved away from having surveillance being the focus to the focus being research… I don’t know [if] there has been a revisiting of the different items that were collected in order to… determine what the core things are that need to be collected year after year and the things that are additional, that for a period of time they add information, and for a period of time, they don’t.* **P14, CDC USA HQ Epidemiology**

ViCo used an approach in which patients were assigned to one of three priority syndromes (i.e., febrile, diarrheal, and respiratory). Stakeholders from UVG reported that the syndromic case definitions changed due to the addition of other research studies. These changes made it difficult to generate compatible and comparable data across the three priority syndromes and over time.

*As time goes by, although we try to keep the system constant, some things change… We cannot necessarily compare the [time] periods… in a way that you can consider… changes in definitions… so that a unit of time, say one year, that worked in the same way, is comparable to another year that worked in a different way… as time went by, [other] variables were being added… [such as] the febrile syndrome. The case definition has depended on the case definitions of the other syndromes.* **P28, UVG Epidemiology, Guatemala**

*We lost many febrile cases because they remained in the other syndromes... It would have been very important not continuing with that exclusion, because then we would have been able to find coinfections… The design of case definitions and the way it is coded… they were only in respiratory, and we stopped capturing a lot of information.* **P22, UVG Laboratory. Guatemala**

Concerns raised around overlapping case definitions and exclusion criteria prompted stakeholders to consider whether ViCo’s original objectives and intended outcomes to increase capacity within MSPAS and describe disease trends in Guatemala were met. This view was shared by stakeholders who felt ViCo needed to re-focus its scope as not all objectives could be fulfilled.

*I saw a contradiction between… strengthening national surveillance and describing the epidemiology of these syndromes in Guatemala... the point of disagreement is in the case definitions because if we are going to strengthen the national surveillance system, we need the case definitions to be identical or compatible with the national system… Choose one of the two goals; we cannot have both.* **P4, UVG Informatics, Guatemala**

#### Data management and structure of the information system

ViCo was praised for its ability to support MSPAS, particularly the Epidemiology Unit, to detect and collect high-quality data on diseases of public health importance, and their causative organisms. Participants from the MSPAS mentioned that ViCo was considered most valuable in identifying the 2009 H1N1 influenza outbreak, guiding decision-making on the influenza immunization schedule and also in assessing measuring the occurrence of rotavirus post-vaccination.

*More than anything else [ViCo] strengthen[ed] the surveillance of the country … it was very useful for the rotavirus issue, mainly for the vaccine... ViCo has also helped us a lot with the issue of influenza... In fact, we just had the study last year… [about] the southern strains or northern strain, [and] what the country needed.* **P35, MSPAS, Central Epidemiologist, Guatemala**

Despite strengths in high-quality data collection, stakeholders expressed a sense of “information overload” when describing the database and described a learning curve for the end-user given the platform that was used to house the data.

*The [data management] system… is basically… a database of its own that tells you about all the questionnaires… has a lot of metadata… but unfortunately, it’s not a program that people around the world use… it’s designed in UVG. Getting down to actual data management… maybe [is] not for the end-user at a beginning level… Everything is there… every single case… the methods that were used, the branching pattern, everything exists [laughs] in this program that people in general don’t use...it’s just it’s very dense to sift through it.* **P20, UVG Epidemiology, Guatemala**

*There were far too many variables being collected… and made the database cumbersome… ViCo was collecting close to 3,000 individual data points per enrollee, which is excessive and then you run into the second issue of trying to maintain the quality of that data.* **P17, CDC Guatemala Country Office**

Stakeholders from UVG mentioned the need to troubleshoot technology to collect, clean, integrate, and obtain the necessary high-quality data within a reasonable time frame. Throughout the data collection phase, nurses reported on the challenges faced when collecting data. Data managers reported challenges in correcting data errors, missing information, and inconsistencies from the database.

*The problem [was] when the REDCAP program restarted [after shutting down sporadically] ... because you had to be entering the data again and, sometimes, the data was blocked or sometimes lost… This can be difficult in that matter of data management because if something is erased, it can no longer be done... when the system fails.* **P25, UVG Project Nurse, Guatemala**

*Data cleaning was the main problem... for a moment it was done regularly, but then it stopped… and suddenly one takes four-year [old] data, and you start to see a lot of problems, so you have to go back many times to the hospitals, to the nurses who help us find something that did not match…. things that had to be reviewed always, always.* **P2, UVG Laboratory, Guatemala**

As time passed, and as leadership shifted across all levels, the consistency in communication, and the approach were affected.

*Even though the data was shared, it was not as so... frequent… there was a period of time when data was no longer shared because apparently, they were doing a restructuring of all emails because the data they sent was to people who were no longer within the Ministry... in the Ministry, we had a lot of rotation, two or three years that many people rotated… so it was difficult to maintain constant communication.* **P11, MSPAS Central Epidemiology, Guatemala**

#### Local control of data

According to CDC stakeholders, ViCo was credited for signaling community trends in the epidemiology of shigellosis, assessing infection prevention effectiveness in hospitals, and evaluating control measures related to nosocomial diseases. The development of interventions for addressing antimicrobial resistance and antibiotic use in the population were also attributed to data from ViCo.

*[T]here was one particular outbreak of Shigellosis in Santa Rosa area … that was … detected by VICO nurses and worked into the night as people were coming into the clinic with this nausea and vomiting diarrhea syndrome that turned out to be Shigella that was associated with a birthday cake that was served in town, so I think that is an example of an outbreak that was detected..* **P18, CDC Guatemala Country Office**

When asked about using ViCo data for public health decision making, there was agreement that additional steps could have been taken to expand its utility especially at the local level.

*Now, at the local level, I think that very little use has been given to the information… the hospital and the [health] area... when they should have been empowered by the information, they were not. This information is useful for us [at the central level], and for the [researchers] of VICO doing the analyses, “the people of ViCo,” but little did [it] involve our people... and we said, "you have to get involved.”* **P30, MSPAS Central Epidemiology, Guatemala**

Local-level stakeholders from MSPAS pointed to an inadequate feedback mechanism from the project to the healthcare providers as the main challenge for leveraging data for public health action and providing immediate analysis to those charged with investigation and follow-up of potential outbreaks.

*The information…[UVG] handles it… and sends a database, but there was no saying: “There goes the database, what do you think? Hey, what new do you need? What else do you think is necessary?” I asked one of the epidemiologists about the ViCo database: “Oh, yes, I received it,” but, “did you see it, did you evaluate it?” “No, I haven’t had time.” There is no point in receiving a database if you are not interested… it is also encouraging staff... not only to have access, but to use it.* **P34, MSPAS District Director, Guatemala**

Although the information was routinely shared among the higher levels in MSPAS, CDC, and the UVG, communication with the local level was perceived to be fragmented by stakeholders who reported that care providers external to ViCo could not access nor use existing health data in real-time to impact patient care.

*We fell short... It would be very important that the information to be presented at some point, but in a general way… and give it more impact by delivering it to all the doctors and then we the doctors could say, “these are the recommendations that I would have,” right? To be able to replicate the knowledge.* **P7, MSPAS Hospital Director, Guatemala**

As well as mentioning limitations in human resources, the time needed to analyze data, and huge workloads as potential reasons for lower data utilization rates, respondents also identified technical constraints to data use for decision making. They agreed that data analysis was a technological capability that required specialized skills.

*You need not just anyone... even though the data is there, it is needed as a technical part to be able to analyze it, and that is not learned overnight… Maybe that’s one of the reasons why it wasn’t used as much… but rather that they used the reports as managers to see… how many [cases] were from such syndromes...* **P3, UVG Informatics, Guatemala**

*[It has] a lot to do with the capacity of the ministry to analyze data, so most of the ministry had not really gone through epidemiology programs or had a lot of opportunity to analyze data. They were used to looking at reports, but not… to really play with data.* **P38**, **CDC USA HQ Epidemiology**

#### Integration of ViCo within MSPAS

Across all stakeholder groups, ViCo was perceived to be a valuable research platform that developed surveillance and laboratory capacity in Guatemala, nationally and at the health facility level. Another notable positive observation among stakeholders was the training provided to a range of staff within MSPAS in outbreak investigation and response, surveillance data collection, and research dissemination.

*[ViCo] provided research capacity for laboratory diagnostics during health emergencies … It served as a platform for outbreak detection, outbreak investigation and response.* **P38, CDC USA HQ Epidemiologist**

Aside from the strengthening local capacity, stakeholders described ViCo as resource-intensive, expensive, and time-consuming and questioned whether it could be replicated and sustained by MSPAS. Given the human resource and financial limitations within MSPAS, stakeholders acknowledged ViCo as a project necessarily supported by external project-hired staff and funding. These limitations impacted the sense of initiative and ownership felt by MSPAS staff.

*As a country… ViCo… is very difficult to replicate… ViCo had personnel exclusively for epidemiological surveillance. They were dedicated exclusively to that... here, we have staff who are dedicated to three or four different things… we don't have the digital part that is much easier… like having an electronic survey, which can be emptied in a database.* **P32, UVG, Epidemiology, Guatemala**

Among stakeholders, ViCo was considered a parallel, independent system designed to feed data to CDC, MSPAS, and health centers but was not integrated within the national health information system. Disparate data collection systems, database interfaces, and data formats complicated the consolidation of data across systems, which according to stakeholders from MSPAS, produced inconsistencies and made it difficult for staff to analyze data.

*There was coordination with the National Epidemiology Center, with the health area and the services, and, weekly, we received the feedback from the database… but, it was a platform that was run by [UVG]. It w’sn't a database that was run by the Ministry of Health… As an epidemiologist in the health area, I could not include the ViCo information in SIGSA, the Health Management Information System... In other words…we always have a parallel information system. Never integrated.* **P34, MSPAS, District Director, Guatemala**

*At some point, the surveillance was completely in the hands of ViCo, and… did not enter our surveillance… it was important to coordinate more to have the information, both ViCo and the project, and the Ministry of Health.* **P11, MSPAS Central Epi, Guatemala**

Staff turnover and limited resources were also mentioned as major obstacles that limited the incorporation of ViCo into the national system. Although ViCo provided the opportunity to transfer capacities to MSPAS, this exchange was perceived by stakeholders as neither consistent nor sustainable.

*Understanding how the Ministry works… there is a lot of personnel turnover and little availability of resources. So, almost all activities that are done that generate new capacities, or improve capacities in the Ministry, are temporary due to the very nature of the Minis’ry's operation.* **P28, UVG Epidemiology, Guatemala**

#### Improvement of the operational and design aspects of ViCo across system, process, and output levels

Stakeholders provided recommendations for strengthening future surveillance projects in Guatemala (Table 2) across system, process, and output levels.

**Table 2:**
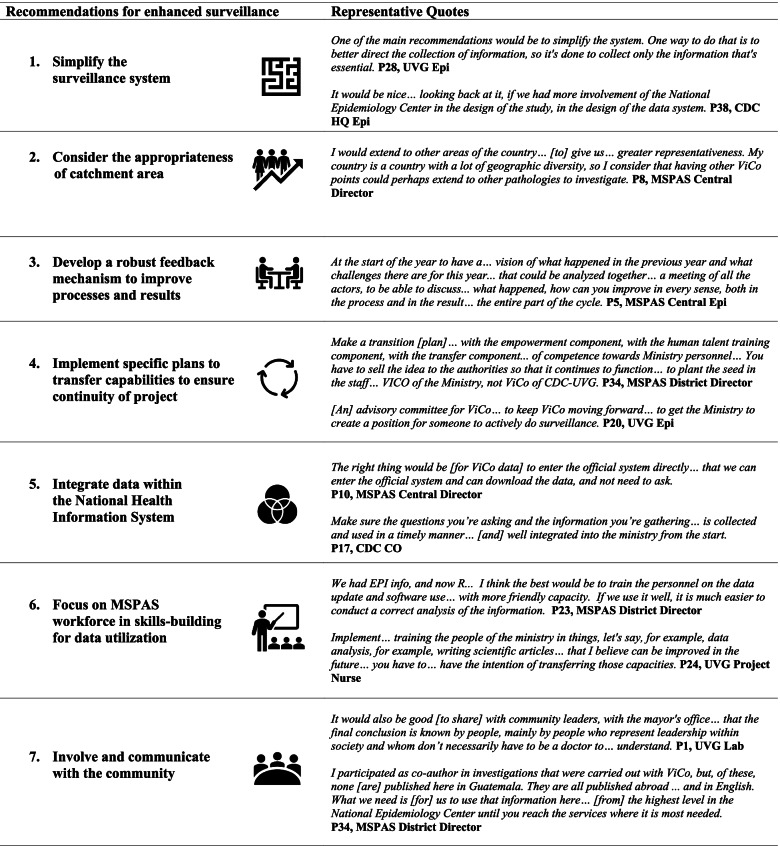
Stakeholder recommendations and representative quotes

### System-level recommendations

#### Simplify the surveillance system

A clear recommendation was to focus the surveillance system’s design on the needs of MSPAS by reviewing and ensuring case definitions, laboratory turn-around time, data elements, survey tools, and data platforms align with Ministry systems, capacities, and expectations.

*One of the main recommendations would be to simplify the system. One way to do that is to better direct the collection of information, so it’s done to collect only the information that’s essential.* **P28, UVG Epi, Guatemala**

#### Consider the appropriateness of catchment area

Suggestions on the representativeness of the geographical area and pathogens vis-à-vis the objectives of surveillance were provided to ensure data are representative of a catchment population to facilitate generalization where this is a priority.

*I would extend to other areas of the country… [to] give us… greater representativeness. My country is a country with a lot of geographic diversity, so I consider that having other ViCo points could perhaps extend to other pathologies to investigate.* **P8, MSPAS Central Director, Guatemala**

### Process-level recommendations

#### Develop a robust feedback mechanism to improve processes and results

According to stakeholders, a strong feedback mechanism could support the continued development of surveillance system management and organizational structure while providing iterative evaluation on data sharing and customization based on surveillance needs. Increased interaction could be nurtured by having more dedicated staff members who hold a stake in the overall system and its direction.

*At the start of the year to have a… vision of what happened in the previous year and what challenges there are for this year... that could be analyzed together… a meeting of all the actors, to be able to discuss... what happened, how can you improve in every sense, both in the process and in the result… the entire part of the cycle.* **P5, MSPAS Central Epi, Guatemala**

#### Implement specific plans to transfer capabilities to MSPAS to ensure continuity of project

Additional collaboration with national authorities (preferably from the formative stages of the project) to concretely institutionalize components of the surveillance system within the MSPAS, considering financial and human resource constraints was also endorsed by respondents.

*Make a transition [plan]… with the empowerment component, with the human talent training component, with the transfer component... of competence towards Ministry personnel… You have to sell the idea to the authorities so that it continues to function… to plant the seed in the staff… ViCo of the Ministry, not ViCo of CDC-UVG.* **P34, MSPAS District Director, Guatemala**

#### Integrate data within the national health information system

Working with MSPAS staff to integrate data within the national health information system, either through add-ons or modifications to the platform, or transfer the database and server to the ministry website could be beneficial according to stakeholders.

*Make sure the questions you’re asking and the information you’re gathering… is collected and used in a timely manner… [and] well integrated into the ministry from the start.* **P17, CDC Guatemala Country Office**

### Output-level recommendations

#### Focus on MSPAS workforce in skills-building for data utilization

Participants across all stakeholder groups recommended additional training opportunities for data analysis, interpretation, and visualization with the national Epidemiology Department and facility level to ensure engagement with the project and real-time data dissemination.

*Implement… training the people of the ministry in things, let's say, for example, data analysis, for example, writing scientific articles… that I believe can be improved in the future… you have to… have the intention of transferring those capacities.* **P24, UVG Project Nurse, Guatemala**

#### Involve and communicate with the community

Dissemination of information at the local level to raise awareness of surveillance system findings was encouraged by stakeholders. The promotion of a more participatory approach with a broad range of participants, including those actively responsible for data collection, could generate trust and a stronger sense of ownership.

*It would also be good [to share] with community leaders, with the mayor's office… that the final conclusion is known by people, mainly by people who represent leadership within society and whom don’t necessarily have to be a doctor to… understand.* **P1, UVG Lab, Guatemala**

## Discussion

This study provides qualitative examples of stakeholder perspectives about the usefulness of a decade-long surveillance project in Guatemala, addresses challenges to its implementation and recommends ways forward. We summarized views, assumptions, expectations, and feelings regarding ViCo’s impact. While all stakeholders acknowledged multiple positive aspects of the project, perspectives varied depending on participant role, knowledge, engagement with the surveillance system, and the length of their involvement. Emergent themes included key strengths related to the detection and collection of high-quality data on various diseases and their etiologies; key challenges related to the size and complexity of ViCo, data management, and limited use of data for public health decision-making. Recommendations focused on the need for simplification, training on data utilization and interpretation, and integration with national systems.

This evaluation highlights some of the potential tensions between routine (i.e., ministry of health-run) and enhanced (i.e., external non-host government funding) surveillance platforms [28]. Enhanced surveillance efforts, such as ViCo, bring additional staff, diagnostic resources, and more detailed design criteria than routine surveillance efforts. More extensive diagnostics are performed in the hope that understanding the detailed causes of illnesses in the area under enhanced surveillance will shed light on what may be occurring in other areas where enhanced diagnostics are unavailable. Over time, stakeholders should revisit the project's fundamental principles when considering making changes to the surveillance system or adding attractive ancillary studies. In an assessment to strengthen Ethiopia's maternal death surveillance and response system, researchers suggested adequate supervisory support from the start to ensure the system became embedded within the health system as a routine practice rather than perceived as a stand-alone activity [29]. Our evaluation generated similar results, as evidenced by stakeholder recommendations to focus additional efforts on integration with MSPAS systems. When enhanced surveillance expertise is housed outside a vertically organized surveillance system, stakeholder roles, responsibilities, and expectations may become fragmented and goals understood differently [29, 30].

Our results emphasize that all details can be crucial to how a system is understood and utilized–including progress towards accepted data standards and guidelines, particularly for how data are cataloged, and case definitions used over time to ensure consistency. As echoed by participants from the health facilities that expressed challenges to the timely access and use of data at the local level, a feedback structure would encourage more dialogue and opportunities for improving the system to meet the surveillance needs of local facilities and the Ministry of Health. This same mechanism could also help define a clear transition strategy for sustaining enhanced surveillance functions once program goals are met. A qualitative assessment of data management and reporting systems in Botswana found that it was essential to build feedback loops into the system, not only through epidemiological bulletins, but also by defining and determining appropriate actions for the investigation and control of outbreaks and measuring progress towards surveillance targets [31]. These strategies would focus on reducing parallel structures, long-term external dependency, and strengthening the existing system, while ensuring that the immediate successes and long-term impacts of enhanced surveillance are not jeopardized [30, 32, 33].

Restricting enhanced surveillance systems to only methods and materials manageable with local resources will not solve the problems of inadequate identification of the causes of illness and death [34]. Instead, there needs to be an a priori plan to extend learning from enhanced surveillance to the broader system and facilitate inclusive analysis of enhanced surveillance data [31, 35–41]. Our findings advocate that transferring knowledge and equipment is not enough to strengthen health systems. In our evaluation, data sharing was particularly impacted by staff turnover and rotations across the facility and national levels. Continuous staff rotation impacted the sense of morale and ownership felt among stakeholders. A One-Health evaluation of the Southern African Centre for Infectious Disease Surveillance found similar operational challenges, citing a lack of resources attributed to information and data sharing, and institutional memory [35].

Further steps to integrate supervision, mentoring and collaboration within the project aims to assure that the expertise generated extends to ministry staff and health workers external to the project are required. Surveillance systems do not serve their purpose when data are not analyzed and interpreted on a consistent basis [42]. As expressed by stakeholders in our study, data utility, data visualization, and data analysis were crucial components that were necessary for an effective disease surveillance system, as it guided decision-making and helped identify and contain outbreaks. Staff views about the quality of a communicable disease surveillance system in Sudan stressed the importance of simplicity in the structure and design of a surveillance system for generating data that can inform decision-making [43]. Similarly, in a study on data-informed decisions in high HIV-prevalence settings in South Africa, inefficient use of information was attributed to the organizational culture and limited capacity of program and facility managers to analyze, interpret, and use information – highlighting the training needs for surveillance [44]. To address this gap, researchers and government leaders tasked with managing surveillance systems have focused on developing local infrastructure to build more effective surveillance and response systems with local motivation and centralized support [36, 41, 45, 46]. Digital health interventions can also be leveraged to facilitate training, supervision, communication, and feature components of performance feedback to promote further engagement of ministry staff in routine data utilization and evaluation [47, 48]. Future surveillance projects will benefit from a comprehensive assessment of skill gaps and identification of opportunities to foster professional growth locally as part of the partnership with local and national staff. Further evaluation is warranted to determine the most effective continuing education approaches to strengthen the surveillance public health workforce.

### Limitations

This study has several limitations. One limitation is the potential for recall and response bias particularly from respondents who were involved with ViCo in the distant past. The information shared by stakeholders was based on personal experiences and subjective impressions. To minimize social desirability bias (a tendency to present reality to align with what is perceived to be socially acceptable) a consistent interviewer was used who was external to ViCo and who could build rapport with a broad base of respondents across agencies and positions to elicit more honest responses about challenges and gaps. Most participants interviewed had moved on to other positions and had little incentive to steer the project’s future direction. These interviewees were not currently involved with ViCo so their opinions may not reflect the current use of ViCo. Another limitation was that we were unable to collect quantitative indicators such as trends in funding for health, diarrheal diseases, respiratory diseases, febrile illnesses, time and use of staff on ViCo and response/control activities, and trends in the number of samples processed. Inclusion of such variables would have provided an additional sense of the monetary investment in the surveillance system and added complementary information to our theme of integration within MPSAS. This assessment nonetheless adds to a growing body of literature on surveillance system evaluations to identify areas for improvement, promote policy changes, and inform future interventions.

## Conclusion

This evaluation has identified system-wide, process, and output challenges related to the delivery, capacity, and management of a surveillance system implemented as a collaborative research effort. These lessons learned may help focus more effective system design for ViCo and similar surveillance projects. To promote surveillance system development, it is essential that data collection is efficient, and provides timely information for public health decision-making. Training in applied epidemiology and data analysis must be an important component of a surveillance system to have capable and empowered personnel to use data, and data use should be a primary evaluation component. Ongoing evaluation of surveillance will be essential to ensure activities meet countries' needs and generate the data needed to plan and implement effective public health policies and strategies not only in Guatemala but in other countries as well. Future assessments should consider economic evaluations to support the non-monetary findings in this study, critical for policymakers who need to make decisions based on limited funding streams.

### Disclaimer

The findings and conclusions in this report are those of the author(s) and do not necessarily represent the official position of the Centers for Disease Control and Prevention.

## Supplementary Information

**Additional file 1.**

**Additional file 2.**

## Data Availability

The interview tools and coding framework are available from the corresponding author upon reasonable request and with permission from CDC.

## Footnotes

1 § §See e.g., 45 C.F.R. part 46, 21 C.F.R. part 56; 42 U.S.C. §241(d); 5 U.S.C. §552a; 44 U.S.C. §3501 et seq.
