# Supplementary figures and images for: Evaluation of the collaborative integrated surveillance system (ViCo) in Guatemala: a qualitative study on lessons learned and future perspectives
Source: BMC Public Health. 2022 Feb 18;22:350. doi: 10.1186/s12889-022-12719-7 (PMC8857857; doi:10.1186/s12889-022-12719-7)

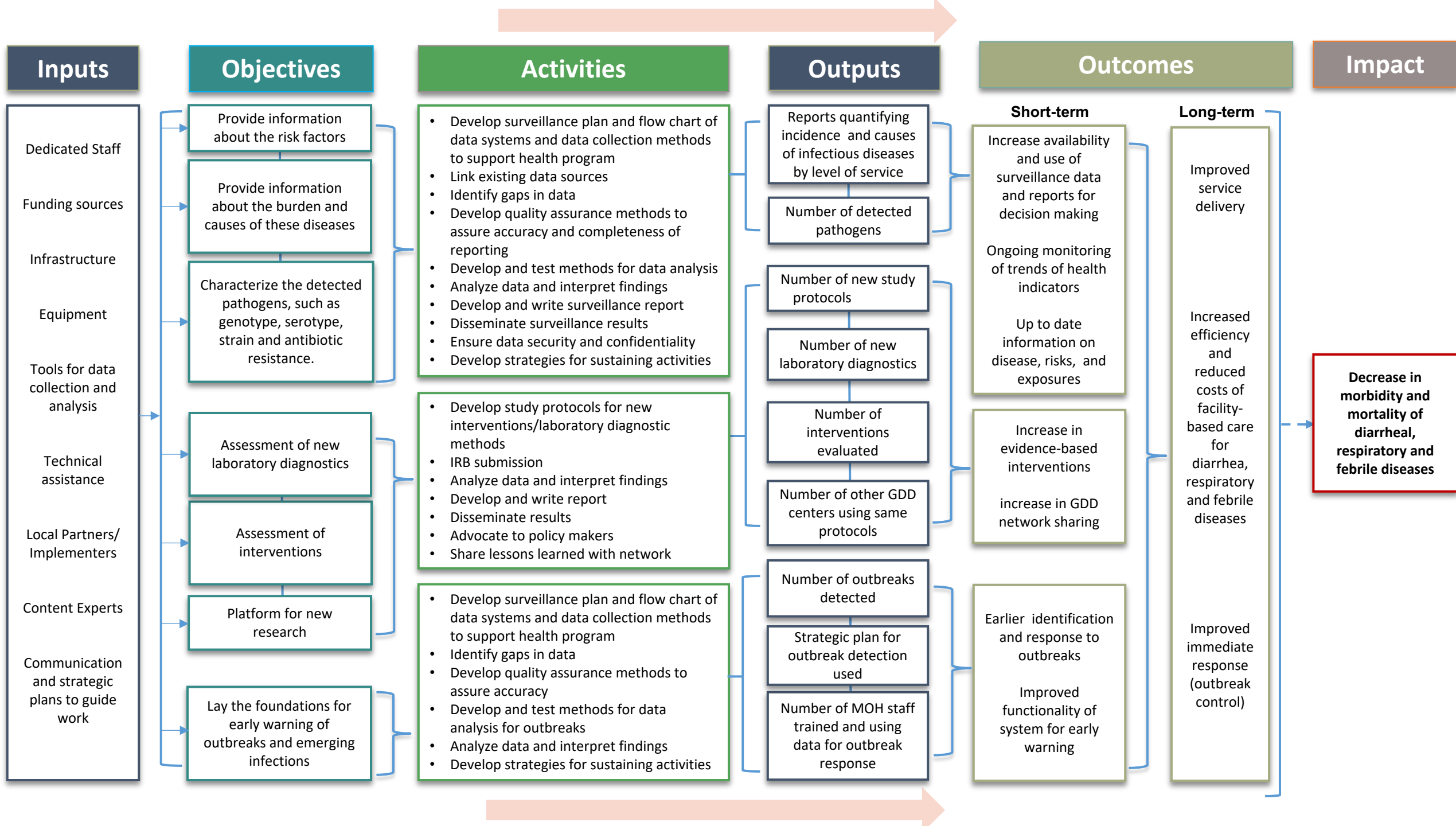

Supplement: Supplementary file 1 — Additional file 1. [file 12889_2022_12719_MOESM1_ESM.pdf]
