# Supplementary material for: Evaluation of the collaborative integrated surveillance system (ViCo) in Guatemala: a qualitative study on lessons learned and future perspectives
Source: BMC Public Health. 2022 Feb 18;22:350. doi: 10.1186/s12889-022-12719-7 (PMC8857857; doi:10.1186/s12889-022-12719-7)
Supplement: Supplementary file 2 — Additional file 2. [file 12889_2022_12719_MOESM2_ESM.docx]

APPENDIX 1

**VICO Interview guide**

**DEMOGRAPHIC DETAILS QUESTIONNAIRE**

**Please answer the following questions in the spaces provided, circle or tick the most appropriate options.**

1. Are you: (please tick as necessary) □ Male □ Female
2. What is your professional background?

□ Epidemiologist

□ Nurse

□ Medical officer

□ Other: (please describe) __________________________________

1. What is your role or job title? _______________
2. How many years of experience have you had in this current job?

□ <1 Year □ 1-2 Years

□ 2-5 Years □ 5-10 Years

□ >10 Years

1. Have you used VICO? (If yes, go to 7)

□ YES □ NO

1. For how many years have you used VICO?

□ <1 Year □ 1-2 Years

□ 2-5 Years □ 5-10 Years

1. In what capacity have you worked with VICO?

□ Central Level epi/surveillance MOH staff □ Regional or district surveillance officer

□ Program managers at national level □ Public health laboratory personnel

□ CDC HQ SME □ CDC Country staff

□ CDC Implementing partner □ Other______________________

***Thank you for taking the time to complete this questionnaire***

**INTERVIEW GUIDE**

**Facilitator’s welcome, introduction and instructions to participants**

**Welcome** and thank you for volunteering to take part in this interview. You have been asked to participate as your point of view is important. I realize you are busy and I appreciate your time.

**Introduction:** This interview is designed to assess your current thoughts and feelings about the VICO surveillance system. The interview will take no more than two hours. May I tape the discussion to facilitate its recollection ? (if yes, switch on the recorder)

**Anonymity:** Despite being taped, I would like to assure you that the discussion will be anonymous. The tapes will be kept safely in a locked facility until they are transcribed word for word, then they will be destroyed. The transcribed notes of the interview will contain no information that would allow individual subjects to be linked to specific statements. You should try to answer and comment as accurately and truthfully as possible. If there are any questions or discussions that you do not wish to answer or participate in, you do not have to do so.

**Informed Consent form: (Go over informed consent form with participant)**

**Introduction**

I would like to start by giving everyone a brief summary of the VICO surveillance system.

The collaborative integrated surveillance system (VICo) in Guatemala for three acute infection syndromes: diarrheal, respiratory and febrile; is a collaboration of the Ministry of Public Health and Social Assistance (MOH), represented by the National Epidemiology Center, The Center for Health Studies of the Universidad del Valle de Guatemala (UVG), and the Centers for Disease Control and Prevention of the United States (US CDC). Surveillance is carried out in Santa Rosa (since 2007) and Quetzaltenango (since 2009) Departments in community and hospital areas of public health services. The objectives of this surveillance are to provide information about the burden and causes of these diseases and lay the foundations for early warning of outbreaks and emerging infections, and assessment of interventions.

Specific Objectives of VICo surveillance system:

- To estimate the incidence of three syndromes of infectious diseases (diarrhea, respiratory and febrile diseases) by level of service (hospital, clinic), age group, etiology, time period and geographical area.
- To evaluate the local impact on public health of prevention and control programs for infectious diseases.
- To identify factors of demographic, socioeconomic, clinical and environmental risk associated with the syndromes, infection with certain pathogens, or the outcome or severity of disease.
- Early detection of a local increase in the number of cases of infectious diseases, which may indicate an outbreak or epidemic, to support rapid response for prevention
- To characterize the detected pathogens, such as genotype, serotype, strain and antibiotic resistance.
- To introduce and evaluate new laboratory diagnostic methods in Guatemala to describe the epidemiology of emerging pathogens.
- To create a platform for potential health research, that would be described in other specific protocols.

Our purpose today is to gain your perspective on the impact of the VICO system as well as to learn from you about the key lessons from VICO

**Warm up**

- First, I’d like to introduce myself. [introduce yourself]. Can you tell us your name?

**Guiding questions**

**Objective of VICO/Evolution of VICO**

1. What do you see as the purpose of the VICO system?
2. Can you list and describe the objectives of the system?
3. Which of these are the most important to you? Why?
4. How have these objectives evolved over time?
5. Can you describe the components of VICO? For example, what is the target population, time period, data sources, what/how data are collected? (If yes) Please describe:
6. Can you describe how VICO generates information that is integrated into national surveillance systems?
7. Is VICO integrated into other systems?
   1. (If yes) Can you describe how VICO is integrated with other surveillance systems?
   2. (If no) How can VICO be better integrated into other surveillance systems
8. Can you please draw a flow chart of the system?
   1. (If yes) Please draw it out.

**Usefulness of VICO**

1. Have you used data from VICO?
   1. (If yes,) How have you used the data from VICO?
2. What did you use the data for?

(Note to facilitator: Description of common activities are presented below)

- Detecting diseases, injuries, or adverse or protective exposures of public importance in a timely way to permit accurate diagnosis or identification, prevention or treatment, and handling of contacts when appropriate.
- Providing estimates of the magnitude of morbidity and mortality related to the health-related event under surveillance, including the identification of factors associated with the event.
- Detecting trends that signal changes in the occurrence of disease, injury, or adverse or protective exposure, including detection of epidemics (or outbreaks).
- Permitting assessment of the effect of prevention and control programs.
- Leading to improved clinical, behavioral, social, policy, or environmental practices and policies; and
- Developing research intended to lead to prevention or control.

1. How useful was this data? (Useful or not useful)
2. If not useful, how could it be rectified?
3. What recommendations can you give to improve VICO and the data ouputs?
4. What data elements were the most useful?
5. Which ones were the least useful?
6. What are your thoughts on the format?
7. What are your thoughts on the content?
8. What are the main issues around actually using the VICO data?
9. What are the barriers to using the VICO data? What are the enablers?
10. When thinking back to how VICO and reports were introduced to you, are there ways that could have been introduced to make it easier/better for you?
11. Did you feel comfortable using VICO data? Do you think there is a need for training? (if yes, explore who would need training, how and where?)
12. How would you make it easier to use/implement VICO?

**Quality of the outputs**

1. Has VICO provided a scientific surveillance report that covers all diseases/health issues under surveillance? It is useful? Why or why not?
2. Has VICO provided interpretations and recommendations of the surveillance results, if appropriate? Were they useful? Why or why not?
3. Have you used VICO to produce scientific publications based on the produced information or activity of the network? Why and why not?
4. Have you used VICO to inform national laws? Why and why not?
5. Have you used VICO data to inform policies development or revisions? If yes, how so? If no, what are your reasons that prevented you from using VICO data to inform policy?
6. Have you used VICO data to evaluate disease interventions? If yes, how so? If no, what are your reasons that prevented you from using VICO data for this purpose?

Have you used VICO data to detect and response to [disease] outbreak? If yes, how so? If no, what are your reasons that prevented you from using VICO data for this purpose?

1. Have you used VICO data to manage a disease pandemic? If yes, how so? If no, what are your reasons that prevented you from using VICO data for this purpose?
2. Have you used VICO data detection and response to [disease] outbreak? If yes, how so? If no, what are your reasons that prevented you from using VICO data for this purpose?
3. Have you used VICO data to plan routine health interventions? If yes, how so? If no, what are your reasons that prevented you from using VICO data for this purpose?
4. Have you used VICO data to inform policies development or revisions? If yes, how so? If no, what are your reasons that prevented you from using VICO data for this purpose?
5. Have you used VICO data to inform MOH financial plans? If yes, how so? If no, what are your reasons that prevented you from using VICO data for this purpose?
6. Do you believe that VICO data is properly reaching the key target groups for the information? How can we improve on reaching the key target groups
7. How can we improve the data presentation?
8. Do you receive the data outputs and reports in a timely manner?
9. How often would you like the VICO outputs and reports provided to you?
10. What is the easiest method to provide you this information?
11. Is it possible to obtain the same data from another source?
12. Do you prefer VICO to these other sources? If yes, why? If no, why do you prefer these other sources?

**Concluding question**

- Of all the things we’ve discussed today, what would you say are the most important issues you would like to express about VICO?

**Conclusion**

- Thank you for participating. This has been a very successful discussion
- Your opinions will be a valuable asset to the study
- We hope you have found the discussion interesting
- If there is anything you are unhappy with or wish to complain about, please contact the local PI or speak to me later
- I would like to remind you that any comments featuring in this report will be anonymous
- Before you leave, please hand in your completed personal details questionnaire
